# Tartary Buckwheat Grain as a Source of Bioactive Compounds in Husked Groats

**DOI:** 10.3390/plants12051122

**Published:** 2023-03-02

**Authors:** Ivan Kreft, Aleksandra Golob, Blanka Vombergar, Mateja Germ

**Affiliations:** 1 Biotechnical Faculty, University of Ljubljana, Jamnikarjeva 101, SI-1000 Ljubljana, Slovenia; 2 Nutrition Institute, Tržaška 40, SI-1000 Ljubljana, Slovenia; 3 The Education Centre Piramida Maribor, Park mladih 3, SI-2000 Maribor, Slovenia

**Keywords:** *Fagopyrum tataricum*, *Fagopyrum esculentum*, flavonoids, rutin, quercetin, food, grain, groats, metabolites

## Abstract

Tartary buckwheat (*Fagopyrum tataricum* Gaertn.) originates in mountain regions of Western China, and is cultivated in China, Bhutan, Northern India, Nepal, and Central Europe. The content of flavonoids in Tartary buckwheat grain and groats is much higher than in common buckwheat (*Fagopyrum esculentum* Moench), and depends on ecological conditions, such as UV-B radiation. Buckwheat intake has preventative effects in chronic diseases, such as cardiovascular diseases, diabetes, and obesity, due to its content of bioactive substances. The main bioactive compounds in Tartary buckwheat groats are flavonoids (rutin and quercetin). There are differences in the bioactivities of buckwheat groats obtained using different husking technologies, based on husking raw or pretreated grain. Husking hydrothermally pretreated grain is among the traditional ways of consuming buckwheat in Europe and some parts of China and Japan. During hydrothermal and other processing of Tartary buckwheat grain, a part of rutin is transformed to quercetin, the degradation product of rutin. By adjusting the humidity of materials and the processing temperature, it is possible to regulate the degree of conversion of rutin to quercetin. Rutin is degraded to quercetin in Tartary buckwheat grain due to the enzyme rutinosidase. The high-temperature treatment of wet Tartary buckwheat grain is able to prevent the transformation of rutin to quercetin.

## 1. Introduction

Tartary buckwheat (*Fagopyrum tataricum* (L.) Gaertn.) originates in regions of South-western China, and is cultivated in China, Korea, northern parts of India, Bhutan, and Nepal [1,2]. It is also traditionally cultivated in several European countries (Luxembourg, Germany, Italy, and Slovenia). In Bosnia and Herzegovina, Tartary buckwheat is cultivated as a mixture of Tartary buckwheat and common buckwheat (*Fagopyrum esculentum* Moench). Recently, Tartary buckwheat has been cultivated in Sweden. Generally, common buckwheat is cultivated more widely than Tartary buckwheat in Asian countries (China, Korea, Japan, India, Nepal, Bhutan, and Pakistan), Central and Eastern Europe (mainly in Russia, Ukraine, Belarus, Kazakhstan, Estonia, Latvia, Lithuania, Poland, Czech Republic, Slovenia, Croatia, Serbia, Italy, and France), Southern and Eastern Africa (mainly Tanzania), Australia (mainly in Tasmania for exporting to Japan as Tasmania soba), Canada, USA, and Brazil [3,4,5].

Buckwheat is used to produce flour dishes, often mixed with wheat flour for bread, pasta, and pancakes. In Central and Eastern Europe, there are traditionally popular dishes made from groats, i.e., husked buckwheat grain. Newly harvested and freshly husked common buckwheat grain is greenish; after time in storage, it becomes light yellow and eventually red. If common buckwheat grain is precooked before husking and/or toasted after husking, it is called kasha. This reddish-brown kasha grain has a nutty flavor. Husked Tartary buckwheat is used in China for making vinegar and beer and in Japan for making tea. Common buckwheat is used in Japan for making liquor. In Korea (the Kangwon area), Tartary buckwheat sprouts are a popular vegetable [6]. Buckwheat husks (after husking the grain to obtain groats) are traditionally used in Japan and China for making cushions. Common buckwheat is a rich source of honey; it is dark, with a unique taste, and is a rich source of antioxidants. Tartary buckwheat is a source of nectar only for small wild insects, but does not attract bees. Thus, fields of Tartary and common buckwheat are important to feed wild insects to maintain their biodiversity. 

Zhang et al. [1] discussed how Tartary buckwheat has high nutritional value, including high protein content and quality in regard to balanced amino acid composition [7,8], and very high levels of antioxidants such as rutin [9]. Rutin and quercetin are flavonoids known for their ability to strengthen blood vessels, and they provide many other potential health benefits, such as a reduction in cholesterol levels and blood clots [10,11]. There is a large difference in the concentration of flavonoids between Tartary buckwheat and common buckwheat. It was found that Tartary buckwheat has higher (at least two-fold) contents of 61 flavonoids and 94 nonflavonoid secondary metabolites than common buckwheat. It is reported that Tartary and common buckwheat grain are rich in secondary metabolites beneficial to human health; among them are nonflavonoid metabolites that also contribute to Tartary buckwheat’s higher health-promoting value in comparison to common buckwheat [12,13].

The goal of this review is to focus on the bioactive compounds of Tartary buckwheat groats, including the impact of mechanical, thermal, and hydrothermal treatments during food preparation on the transformation of compounds. 

## 2. Synthesis of Bioactive Substances in Tartary Buckwheat

The ancestors of today’s Tartary buckwheat were plants that were consumed by herbivorous dinosaurs. About 85 million years ago [1], the common ancestors of today’s sugar beet and Tartary buckwheat were among the herbs available during the time of the dinosaurs. 

**Figure 1:**
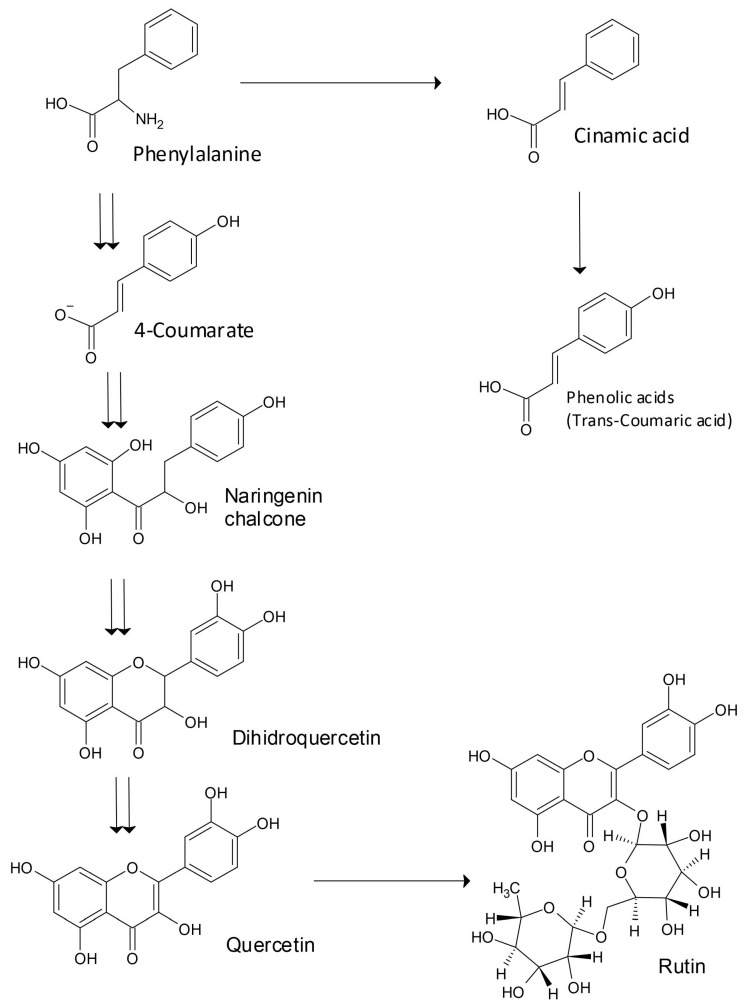
Basic biosynthesis pathway for the synthesis of flavonoids rutin and quercetin, and phenolic acids.

Several genes important for the regulation of quercetin and rutin biosynthesis have been identified [1]. The capability of buckwheat to survive under high levels of abiotic stress is due to the appearance of several gene groups involved in the transfer of signals, the regulation of the activity of genes, and the transfer of molecules. These genetic resources have facilitated the discovery of physiologically and nutritionally important plant genes, and the possibility of genetic improvement in Tartary buckwheat [1,5]. There are 769 gene groups involved in the evolutionary side branching of Tartary buckwheat and sugar beet, establishing the divergence of Caryophyllales before the separation of asterids and rosids [14]. Zhang et al. [1] reported that the ancestors of Tartary buckwheat and sugar beet split from each other about 85 million years ago. The basic biosynthesis pathway for the production of the flavonoids rutin and quercetin and phenolic acids is highlighted in Figure 1. The solar UV-B irradiation of plants may increase the amount of rutin and other UV-absorbing secondary substances in buckwheat grain and the green parts of plants [3,4].

## 3. Technologies for Husking Buckwheat Grain and Preparing Groats

Due to their robust husk, dormant Tartary buckwheat seeds may remain alive in the soil for several years and, under favorable conditions, can be activated. Tartary buckwheat may appear as a weed in other crops and is often a weed in a crop of common buckwheat (*Fagopyrum esculentum* Moench) [4]. 

One method to husk buckwheat is to use non-precooked grain. The raw husked common buckwheat groats are green when newly harvested buckwheat is husked. After some weeks, chlorophyll fades and phenolic substances oxidize, so the older non-precooked buckwheat groats are yellow, and later become reddish. From the green color it is possible to estimate if raw husked groats are fresh and made from newly harvested common buckwheat. By pressing the flat side of the knife, non-precooked groats are broken into floury particles, and when cooking the groats, they become slimy [3,4]. 

**Figure 2:**
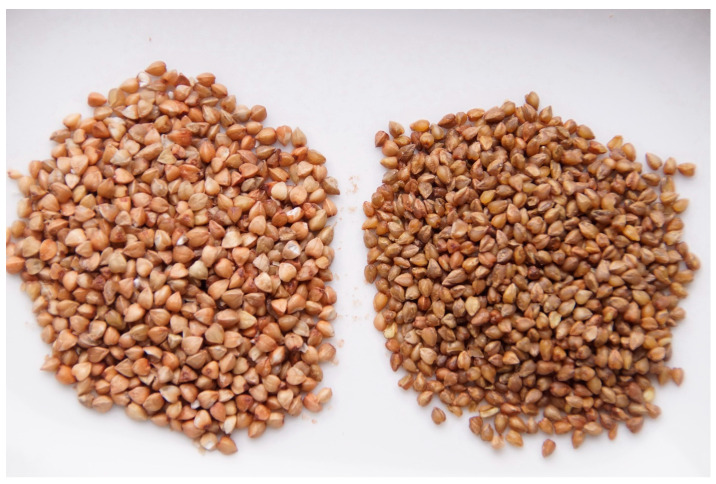
Buckwheat groats obtained using the traditional precooking method: left, common buckwheat; right, Tartary buckwheat kasha (husked buckwheat grain).

Buckwheat groats obtained by precooking are harder, and break under pressure into larger particles with a vitreous appearance (Figure 2). Precooked buckwheat groats (husked buckwheat grain) cook nicely, and they also have a special taste. Substances of traditional buckwheat groats, important for this distinctive taste, have been studied [15]. Buckwheat groats are popular in Slovenia, Croatia, Poland, Belarus, Ukraine, Russia, some parts of China (Shanxi and Shaanxi), and Japan (Shikoku island) [3,4]. They were also once known in Carinthia, Austria. In Karelia (Finland), buckwheat groats cooked in milk or cream are added on the top of traditional open pierogi (karjalan piirakka) on a base of rye dough [3,4]. 

For husking, precooked buckwheat grains are firstly soaked in boiling water, and during this treatment, the starch swells. In doing so, the husk bursts on one of the three corners. When ready, the water is removed and the grain is dried at a moderate temperature. In old times, the grain was traditionally dried on canvas in the shade or on a warm top of a wood-burning stove. The grains were repeatedly mixed to dry evenly. They were dried only so that the husk was dry and brittle, and the inside of the grain was elastic, but solid enough not to squeeze or smear when the process continued. Properly dried grain was placed in a husking device called a stope. A stope consists of a stable hollowed trunk with an opening for at least five liters of grain. Above the opening is a fixed beam with a downward-facing iron tip with horizontal ribs. The beam went up several tens of inches and then fell freely into the prepared grain. This was repeated as needed, even a hundred times if necessary. When the grains were struck, the husk was separated from the grain, and groats (kasha) were formed. When most grains were husked, farmers used ventilation to separate light husks from heavier kasha grains. In areas rich in streams, the stopes were powered by water wheels. Stopes were foot-powered in plain landscapes. In Carinthia, Austria, hydrothermally pretreated buckwheat was husked by treading on grain with heavy wooden shoes [16]. Preparing husked buckwheat grain, kasha, is very challenging. The process has been known among Slovenians for a long time, as was described by Valvasor [17]. 

The modern husking of buckwheat grain is essentially the same as it used to be—cooking, drying, husking, drying again, and blowing off the husks. The details of technology are the intellectual property of each producer, especially in husking technology for Tartary buckwheat, which is because of the significant issue of thick husks. To separate the remaining unhusked grain from the husked grain, producers use photocell-supported equipment. Buckwheat kasha obtained the traditional way by precooking before husking has a special taste and properties, including the composition of bioactive substances (Table 1). Tartary buckwheat grains have a much higher content of rutin and total flavonoids than common buckwheat grains (Table 1). In addition, the husked grains of Tartary buckwheat have a much higher content of rutin than the husked grains of common buckwheat. As a result of hydrothermal treatment, husked Tartary buckwheat grains contain much less rutin in comparison to intact grains, but a much higher content of quercetin. This is the result of the enzymatic conversion of rutin to quercetin during hydrothermal treatment.

Phenolic acids and other phenolic metabolites are present in Tartary buckwheat grain at low concentrations in comparison to rutin and quercetin (Table 1) [40,41]. Their content is more substantial in the green parts (leaves) and flowers of buckwheat plants [43]. For example, the concentration of neochlorogenic acid is 0.3% in buckwheat leaves, much higher than its concentration in grain [40,44].

## 4. Interaction among Substances in Buckwheat Groats and Impact on Bioactivity

### 4.1. Impact of Buckwheat Phenolic Substances on Proteins and Cholesterol

During husking and the hydrothermal pretreatment of buckwheat grain, phenolic substances have an impact on protein digestibility [45]. Substantial interactions have been reported between proteins and phenolic substances, and as such, the digestibility of buckwheat proteins is reduced. In any way, microbial processes in the large intestine enhance the digestion of proteins [45]. It was established that phenolic substances in buckwheat grain lower the digestibility of proteins. Ikeda et al. [46] reported that tannic acid and catechin significantly inhibit the in vitro peptic and pancreatic digestion of buckwheat globulin. Ikeda et al. [46] and Ikeda and Kishida [47] studied the impact of secondary buckwheat metabolites on the in vitro digestibility of buckwheat grain proteins. It was established that undigested buckwheat proteins can reduce cholesterol levels in serum by increasing the fecal excretion of steroids, which is induced by the binding of steroids to proteins. Ma and Xiong [48] reported that digestion-resistant peptides are largely responsible for the elimination of bile acids and lowering the risk of the appearance of a high level of cholesterol in human serum. In this way, buckwheat proteins, in their interaction with phenolic substances, protect the human cardiovascular system.

### 4.2. Transformation of Phenolic Substances

**Figure 3:**
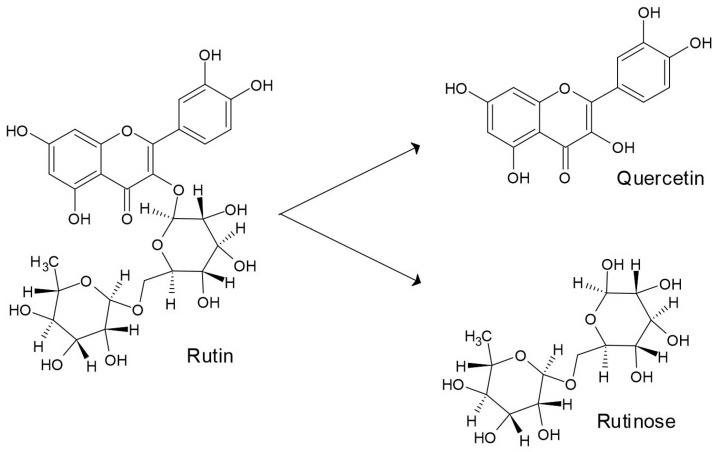
Transformation of rutin to quercetin and sugar rutinose.

The treatment of Tartary buckwheat grain with water impacts the transformation of rutin to quercetin by the rutin-degrading enzyme (Figure 3) [49,50,51,52]. The activity of the rutin-degrading enzyme is prevented when buckwheat grains are exposed to temperatures at about 80 °C or higher during husking treatment [52].

Ingested quercetin can cross the blood–brain barrier and accumulate in the brain tissue [53]. Indeed, important bioactivities have been established for quercetin and its derivatives, not just in blood vessels, muscle, and the gastrointestinal system, but also in the brain. Quercetin and other phenolic metabolites have been isolated from the stool samples of people who had eaten food rich in phenolic substances [53].

Phenolic compounds are often transformed in the gut before their absorption. The gut microbiota are essential in this process [54]. Large-sized dietary phenolic substances are poorly absorbed, while small-sized products of microbial conversion are more easily absorbed in the colon. It is interesting to note the suitability of buckwheat groats for feeding dogs. There are nutritional differences between buckwheat groats that are husked raw and those that are husked after the precooking of the grain. The first are toxic to dogs and cause them liver damage, and the second can be safely consumed [55]. Carnivores are susceptible to low-molecular-weight tannins, but not to high-molecular-weight tannins. Conversion occurs in the case of precooked buckwheat groats, but not in groats obtained without precooking.

### 4.3. Tartary Buckwheat Diet in the Prevention of Diabetes 

The complexation of quercetin in Tartary buckwheat materials with molecules of starch has an influence on the in vitro digestibility and physicochemical properties of starch [56]. The effects of quercetin–starch complexation indicate that food products based on Tartary buckwheat have lower starch digestibility. Indeed, quercetin in Tartary buckwheat can reduce body weight, serum triacylglycerols, and low-density lipoprotein. In rats, a diet with 0.1% quercetin was shown to have the significant impact of lowering low-density lipoprotein concentrations in serum, with no such effects on high-density lipoprotein. Tartary buckwheat has also been shown to prevent increases in body weight and fat deposition during high-fat intake in rats, although on the other hand, this was reported to protect against hepatic stenosis [10,11,57]. A buckwheat diet can also reduce insulin and ameliorate glucose intolerance in humans [58].

Rat experiments with common buckwheat have further suggested the complexity of the impact of the gut microbiota. Indeed, Peng et al. [57] suggested that the link between weight gain and the gut microbiota is very complex, with a need for further studies. Luo et al. [59] studied the slow digestion properties of Tartary buckwheat starch treated with ethanol extract. The slow digestibility of this starch appeared to be due to the impact of phenolic substances on starch. In the in vivo experiments, mice showed reduced postprandial glycemic responses. The data of Luo et al. [59] for Tartary buckwheat grain and glycemic responses were similar to those obtained earlier in common buckwheat [58].

### 4.4. Buckwheat Groats in a Health-Preserving Diet

In Japan, buckwheat groats are less well known than in Europe [3]. However, soup with buckwheat groats (soba-gome) is served in Tokushima on Shikoku island [60]. In China, Tartary buckwheat groats are known in some regions, for example, in Shanxi and Shaanxi [3].

**Figure 4:**
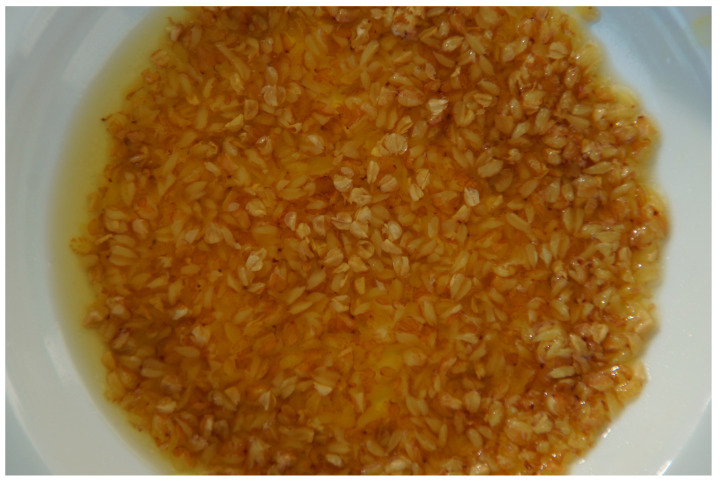
One of the simplest dishes with Tartary buckwheat kasha is Tartary buckwheat soup. About 10 percent (volume) Tartary buckwheat kasha is added to very lightly salted water, boiled for few minutes, and a few spoons of spicy olive oil are added.

Many dishes can be made with common or Tartary buckwheat kasha [61,62]. Some attractive dishes can be made simply by replacing white rice or husked barley with common or Tartary buckwheat kasha (Figure 4). It is possible to cook buckwheat kasha one day, preserve it overnight in the refrigerator, and mix it into oil-fried vegetables (sliced pumpkins, tomatoes, etc.) the next day, or mix it with cottage cheese, cream, and/or walnuts, and/or sliced apples for baking in the oven [61]. In addition to rutin, buckwheat dishes, especially Tartary buckwheat dishes, contain metabolites such as tannins and quercetin, which also inhibit starch degradation [4,63,64]. As a food rich in quercetin, Tartary buckwheat groats may hold nutraceutic potential against SARS-CoV-2 due to their ability to inhibit the virus at various stages of its life cycle [65,66].

### 4.5. Bioactivity Impact of Metabolites

During the treatment of Tartary buckwheat groat, the concentrations of bioactive substances with strong impacts on human health are altered. This includes the concentration of rutin and quercetin in buckwheat groats (Table 1).

The supplementation of rutin-rich diets with vitamin C is able to reduce oxidative stress, have an impact on glycemic stress, and reduce fasting blood glucose in patients with type 2 diabetes [10]. A rutin-rich Tartary buckwheat diet may be effective in reducing body weight through its antioxidant effects [11,13]. The fermentation of dietary fiber from Tartary buckwheat helps to improve its solubility, in addition to the impact of flavonoids on countering obesity [13].

Phenolic substances in buckwheat have significant bioactivities in addition to their antioxidative and anti-inflammatory effects [43]. Phenolic substances incorporated in the hydrophilic erythrocyte membranes are barriers against free radicals. This is possible because of the double nature of phenolic substances: a lipophilic phenolic aglycone and a hydrophilic sugar part. Buckwheat flavonoid extracts have an impact on the antioxidant system in the liver and brain [44]. 

Ingested quercetin and its glycosides are metabolized during human digestion, and are absorbed and transported as conjugates with the blood. A major metabolite of quercetin is quercetin-glucuronide, which is transported to the target tissues. Following the separation of the sugar part of hydrophilic quercetin-glucuronide, the hydrophobic aglycone remains at injured sites to perform the improvement of pathological conditions [53].

High levels of rutin and quercetin in Tartary buckwheat, and high levels of antioxidative impacts, have further effects on the cytotoxic and antigenotoxic impacts [64,67]. A study of the antigenotoxic effects of Tartary buckwheat in human hepatoma cell lines has shown that flavonoid-rich Tartary buckwheat products are more effective for maintaining health in their complexed form than as the single active substances, rutin or quercetin [67]. It has been suggested that the Tartary buckwheat metabolites rutin and quercetin may be effective against cancers, based on experiments in cell lines and animal models for mammary, colon, skin, and other cancers [68,69,70,71,72,73].

## 5. Conclusions and Future Perspectives

The grain and groat of common buckwheat contain a low concentration of rutin, while its concentration in the grain of Tartary buckwheat could be up to 1%. The solar UV irradiation of plants may increase the amount of rutin and other UV-absorbing secondary metabolites in buckwheat grain. 

During the processing of buckwheat grain using hydrothermal treatment to obtain husked product (groats or kasha), there are various possible transformations and interactions among the constituents, including the formation of quercetin, as a degradation product of rutin by rutin-degrading enzyme. The activity of rutin-degrading enzyme is prevented by hydrothermal treatment of buckwheat grain products at about 80 °C or more. 

In Tartary buckwheat groats, quercetin complexation with starch molecules has an impact on the in vitro digestibility of the starch and the appearance of resistant starch, thus altering the physicochemical properties of Tartary buckwheat products. The effects of such flavonoid–starch or flavonoid–protein complexation indicate that food products based on Tartary buckwheat will show lower starch or protein digestibility. Quercetin in Tartary buckwheat can reduce body weight, serum triacylglycerols, and low-density lipoprotein. During digestion in the large intestine, due to protein–phenol interactions of the buckwheat grain, protein digestion is slowed down. However, again, the microbial processes in the colon partly enhance the digestibility of the proteins. Slowly digestible buckwheat groat protein can reduce cholesterol levels in serum by increasing the fecal excretion of steroids. Tartary buckwheat has also been shown to prevent increases in body weight and fat deposition during high fat intake in rats. A buckwheat diet can also reduce insulin and ameliorate glucose intolerance in humans. Important bioactivities have been established for ingested quercetin and its derivatives, not just in blood vessels, muscle, and the gastrointestinal system, but also in the brain. 

Further understanding of the metabolism of rutin and quercetin in buckwheat plants and food products, and how different forms of flavonoids impact the health of consumers, should prove useful in achieving better human health. Hopefully, continuous progress on the wider use of Tartary buckwheat and its products, including groats, over time can fulfill the yield gap needed to feed our global community with a health-preserving diet. 

## Figures and Tables

**Table 1:** Content of major bioactive compounds isolated from buckwheat grain and groats.

Compound Name	Content in Dry Weight						References
	Common Buckwheat Grain	Common Buckwheat Raw Groats	Common Buckwheat Treated Groats	Tartary Buckwheat Grain	Tartary Buckwheat Raw Groats	Tartary Buckwheat Treated Groats	
Flavonoids						16–23 µg/kg in roasted	[18]
Flavonoids				18 mg/g			[19]
Flavonoids	18.8 mg/100 g						[20]
Flavonoids	0.04%			2.04%			[21]
Flavonoids	24.4 µg/mg			142.2 µg/mg			[22]
Flavanols	15–75 mg/100 g						[23]
Flavonols	0–50 mg/100 g						[23]
Rutin						12–18 µg/kg in roasted	[18]
Rutin	76 mg/100 g			1197 mg/100 g			[22]
Rutin	20 mg/100 g			1669 mg/100 g			[21]
Rutin	0.0584 g/100 g			1.83–1.97 g/100 g			[24]
Rutin	12.2–13.6 mg/100 g			1808–1853 mg/100 g			[25]
Rutin	272–341 mg/kg			8060–16,633 mg/kg	5569 mg/kg		[26]
Rutin	115–181 mg/kg	230 mg/kg	87.9 mg/kg				[27]
Rutin	0.1–0.92%			1.19–2.91%			[28]
Rutin	17.2–17.7 mg/100 g						[29]
Rutin	0.8–1.7%						[9]
Rutin	0.39–0.52%			0.87–1.33%			[30]
Rutin				14,600 mg/kg			[31]
Rutin				11,900–21,449 mg/kg			[32]
Rutin		44.2–51.1 mg/100g					[33]
Rutin		0.259 g/kg			80,940 mg/kg		[34]
Rutin					13,610 mg/kg		[35]
Rutin						5 mg/kg	[36]
Rutin	62 mg/kg		52–55 mg/kg	7623 mg/kg		6191–6975 mg/kg	[37]
Rutin				14 mg/g		8 mg/g	[38]
Quercetin				0.04 mg/g		0.07 mg/g	[38]
Quercetin	212 mg/kg						[39]
Quercetin				473–900 mg/kg			[26]
Quercetin		0.002 g/kg			8 mg/kg		[34]
Quercetin						1.9–2.8 µg/kg in roasted	[18]
Quercetin	26 g/kg		22–28 mg/kg	128 mg/kg		54–116 mg/kg	[37]
Caffeic acid	0.012–0.022 mg/g			0–005 mg/g			[40,41]
Chlorogenic acid	0.027–0.090 mg/g						[40]
Neochlorogenic acid	0.092–0.216 mg/g			0.017 mg/g			[40,42]
Coumaric acid	0.006–0.132 mg/g			0.002–0.010 mg/g			[40,41]
Trans-sinapic acid	0–0.008 mg/g						[40]
*p*-hydrobenzoic acid				0.022–0.088 mg/g			[41]
Ferulic acid				0.019–0.073 mg/g			[41]
